# Autoantibodies in the Diagnosis, Monitoring, and Treatment of Membranous Nephropathy

**DOI:** 10.3389/fimmu.2021.593288

**Published:** 2021-03-22

**Authors:** Vladimir Tesar, Zdenka Hruskova

**Affiliations:** Department of Nephrology, 1st Faculty of Medicine, General University Hospital, Charles University, Prague, Czechia

**Keywords:** membranous nephropathy, anti-PLA2R antibodies, anti-THSD7A antibody, remission, outcome

## Abstract

The discovery of anti-podocyte antibodies in primary membranous nephropathy (MN) has revolutionized our approach toward the diagnosis and treatment of this disease. Evaluation of serum levels of anti-podocyte antibodies paved the way for non-invasive diagnosis and helped distinguish between primary and secondary MN although the relationship between anti-podocyte antibodies and cancer remains to be elucidated. Serum levels of anti-PLA2R antibodies directed against the major podocyte autoantigen are related to MN activity and the decrease in serum levels of anti-PLA2R antibodies in response to treatment (immunologic remission) also serves as an early indicator of the later putative proteinuric remission, enabling personalization of the treatment. The serum levels of anti-podocyte antibodies also enable the prediction of renal outcomes in terms of both remission and the risk of progression to end-stage renal disease. The positivity of anti-PLA2R antibodies before renal transplantation is associated with the risk of recurrence of MN. It remains to be established if all these relations observed in patients with anti-PLA2R antibodies are also valid for expanding spectrum of antibodies directed against recently discovered minor antigens (e.g., THSD7A, NELL-1, semaphorin 3B).

## Membranous Nephropathy—a Major Cause of Nephrotic Syndrome

Membranous nephropathy (MN) is defined by the typical histopathological patterns of thickening of the glomerular capillary wall with subepithelial immune deposits gradually embedded in newly formed glomerular basement membranes with positive granular immunofluorescence of IgG and C3 (1, 2). Although kidney biopsy is an invasive procedure, it remains the gold standard for the diagnosis of MN.

More than 80% of patients with MN present with nephrotic syndrome (3). MN is the cause of nephrotic syndrome in approximately 25% of adults (more frequently in males) and is the most common cause of nephrotic syndrome among older adults (4). The glomerular filtration rate at presentation is usually normal or only slightly impaired.

MN may be secondary, most often as one of the histologic classes (class V) of lupus nephritis, or pathogenetically related to cancer, hepatitis B, sarcoidosis, or some drugs (1, 2). However, in about 75% of patients with MN, the primary cause is not apparent, with the disease traditionally classified as idiopathic (until the discovery of anti-podocyte antibodies) or primary MN. Light microscopy does not reliably distinguish between primary and secondary MN. Immunofluorescent deposition of IgG4 is typical for primary and IgG1, IgG2, and IgG3 for secondary MN with C1q often present in membranous (class V) lupus nephritis (1, 2).

Proteinuria and serum creatinine are still used to stratify the renal risk in patients with primary MN (5, 6). Patients with sub-nephrotic proteinuria have good long-term renal outcomes and should not be treated with immunosuppression. On the other hand, predicting renal outcomes in patients with nephrotic proteinuria is much more difficult as it ranges from spontaneous remission [in as much as 50% of patients during longer follow-up (7)] to the progression to end-stage kidney disease [in about 30% of patients within 10 years of follow-up (8)].

Due to the high propensity to spontaneous remission and toxicity of traditional treatment according to KDIGO guidelines (9) immunosuppressive treatment with alkylating agents is initiated only in patients with nephrotic syndrome persisting for at least 6 months, severe complications of nephrotic syndrome, or an increase in serum creatinine by 30% within 6–12 months.

## Expanding Spectrum of Anti-Podocyte Antibodies in MN

The experimental model of MN, Heymann nephritis (10), was induced in its active form by the immunization of the rats with the material derived from the proximal tubule and in its passive form by injecting the rats with heterologous IgG to a crude rat tubular extract (11).

Heymann nephritis and human MN were always suspected to be autoimmune diseases putatively caused by anti-podocyte antibodies. As a proof of the concept in 1995, megalin, expressed by rat podocytes and on the luminal membrane of the cells of the proximal tubules (where it is involved in the uptake of a variety of proteins) was identified as an autoantigen of Heymann nephritis (12).

In humans, megalin is believed to be expressed only by the brush border of the proximal tubule, but not the human podocytes. Therefore, it could not be the target antigen in human MN. Recently, however, primary renal interstitial disease with anti-brush border antibodies and IgG-positive immune deposits along the tubular basement membrane, segmental subepithelial glomerular deposits, subnephrotic proteinuria, and circulating antibodies against megalin (LDL receptor-related protein 2—LRP2) was described (13, 14). Segmental MN in humans with anti-megalin antibodies is apparently due to some (although not as dense as in rats) expression of megalin in human podocytes (14). However, megalin is not the podocyte autoantigen in patients with typical MN.

The role of podocyte proteins in eliciting an immune response in MN was first demonstrated with the finding of neonatal MN in a child of a mother with a mutated MME gene for neutral endopeptidase (NEP), normally expressed by podocytes. During pregnancy, the mother raised antibodies against NEP of the fetus, which crossed the placenta and caused transient MN in the newborn (15).

However, the major podocyte autoantigen in MN was only identified in 2009 as the phospholipase A2 receptor [PLA2R (16)]. Anti-PLA2R antibodies occur with disease presentation in about 70–80% of patients (more often men). The presence of anti-PLA2R antibodies is highly specific for primary MN. However, they may also be present in some patients with secondary MN (sarcoidosis or HBV-associated MN).

Since 2009, several minor podocyte autoantigens have been identified in patients with MN: antibodies against thrombospondin type-1 domain-containing 7A [THSD7A (17)] are present in 3–5% of patients (more often women) (6), antibodies against neural epidermal growth factor-like 1 protein [NELL-1 (18)] are present in 5–10% of patients, and antibodies against semaphorin 3B (19) are present namely in pediatric patients. Anti-NELL-1 antibodies were shown to be much more common in segmental MN (20). Very recently, antibodies against protocadherin 7 (PCDH7) and high termperature recombinant protein A1 /HTRA1), both of IgG4 subclass, were described in several patients with anti-PLA2R-negative primary membranous nephropathy (21, 22).

Additionally, antibodies directed against exostosin-1 and exostosin-2 (EXT-1 and EXT-2) were found to be specific for lupus MN (23) and antibodies against neural cell adhesion molecule-1 (NCAM-1) occur in about 6% of patients with lupus MN, but may also occur in 2% of patients with primary MN (24). In membranous lupus nephritis EXT1/EXT2-positive patients have compared to EXT1/EXT2-negative patients more often nephrotic proteinuria, but less glomerulosclerosis and interstitial fibrosis. EXT1/EXT2-negative patients progressed to end-stage kidney disese more quickly and more frequently (25).

Antibodies to EXT1/EXT2, NELL-1 and Sema3B are of IgG1 subtype and are complement-activating, antibodies to PCDH7 are, similarly as anti-PLA2R antibodies predominantly of IgG4 subtype and do not activate the complement.

Different autoantibodies may thus be associated with different pathogenesis, pattern of histologic damage and different clinical phenotype including response to treatment and outcome (26).

All as-yet identified autoantigens are responsible for up to 90% of cases of idiopathic (primary) MN, so the future discovery of further minor antigens cannot be excluded (27). Approximately 1% of patients with MN may have double positivity for both anti-PLA2R and anti-THSD7A antibodies (28, 29). In a large cohort of 1,012 patients with biopsy-proven MN, anti-THSD7A antibodies were identified in 2.8% of patients, eight of which (0.8%) were double positive for anti-THSD7A and anti-PLA2R antibodies (30).

Double positivity of MPO-ANCA and anti-PLA2R was described in two patients with ANCA-associated glomerulonephritis combined with MN-like lesions (31). In other studies necrotizing/crescentic glomerulonephritis was described in a small proportion (about 0, 3%) of patients with membranous nephropathy (32, 33). Although most of these patients have concomitant ANCA or anti-GBM antibodies this histologic pattern was observed also in several patients with anti-PLA2R antibodies only. It remains unclear if anti-GBM damage caused by ANCA or anti-GBM antibodies may expose podocyte antigens, or, on the other hand, subepithelial deposits in membranous nephropathy may enhance damage to GBM by ANCA or anti-GBM antibodies.

## Glomerular Binding of Anti-PLA2R Antibodies

The binding of anti-PLA2R IgG4 antibodies to podocytes can usually be detected in patients with MN using immunofluorescence or immunohistochemistry. However, it is not always parallel to circulating anti-PLA2R antibodies, if measured at the time of biopsy (34, 35). Although glomerular binding of anti-PLA2R antibodies is highly specific for primary MN, it also occurs in some patients with HBV, sarcoidosis, or cancer-associated secondary membranous nephropathies.

Glomerular binding of anti-PLA2R antibodies may persist for months even after the systemic autoimmune response is suppressed. Alternatively, the absence of circulating anti-PLA2R antibodies is observed in some patients with active MN and glomerular anti-PLA2R binding. It is speculated that the lack of circulating antibody detection could be due to the high avidity of anti-PLA2R antibodies to the podocytes (35).

In some patients with MN positive for glomerular PLA2R staining, circulating antibodies may not be detectable at presentation, but may occur later during follow-up (and treatment) of the disease (36). This seroconversion could be explained by the buffering capacity of the kidney, that is, circulating antibodies occur only after the binding capacity of the kidney is exceeded, emphasizing the importance of the evaluation of glomerular PLA2R staining or repeated evaluation of anti-PLA2R in anti-PLA2R negative patients, especially during the first 6-months of follow-up.

In some patients with positive glomerular staining, circulating anti-PLA2R antibodies may only become detectable at 12 months, or even during late relapse at 28 months (37). There can be even an early absence of glomerular IgG4-anti-PLA2R positive staining, as some patients may have initially IgG1 dominant immune deposits (potentially with another podocyte target antigen), that are PLA2R negative. This suggests putative later histologic conversion (to IgG4-PLA2R staining) which could be demonstrated only through repeat kidney biopsies (38). Positive glomerular staining of anti-THSD7A (39) and anti-NELL-1 antibodies (18) was also described in patients with MN with autoantibodies directed to these minor autoantigens, respectively.

## Anti-Podocyte Antibodies in the Pathogenesis of MN

The role of complement in the pathogenesis of MN was demonstrated by the deposition of C3 and C5b-9 in both Heymann nephritis and human MN. Moreover, glomerular injury in Heymann nephritis can be prevented by blocking the formation of the C5b-9 complex on the podocyte membrane (40).

Although the direct pathogenic role of anti-podocyte autoantibodies in MN has been supported by the observation in passive Heymann nephritis induced in rats by injection of IgG against tubular extract (11), in humans, it was difficult to reconcile the preponderance of non-complement activating IgG4 anti-PLA2R antibodies (at least at a later course of anti-PLA2R antibody-positive MN) with the well-documented complement activation. One putative explanation could be the activation of the alternative complement pathway in MN recently demonstrated using proteomic analysis of microdissected glomeruli (41), which may be preceded by earlier glomerular deposition of other IgG subclasses that activated the classical complement pathway (35). The recently described anti-NELL-1 antibody is predominantly of the IgG1 class and maybe the cause of prominent C3 deposits (40). The role of complement regulatory proteins in the pathogenesis of MN also remains to be elucidated (42).

THSD7A (unlike PLA2R1) is expressed in human and murine podocytes. As a result, it was possible to show that administration of human anti-THSD7A antibodies to mice results in their binding to murine podocytes with subsequent foot process effacement, histologic changes typical of MN and proteinuria (43). However, in this model, proteinuria preceding complement deposition was only small and transient (44).

The pathogenic role of anti-PLA2R antibodies was recently demonstrated in a mouse model in which the major target antigen, human PLA2R, was expressed in mouse podocytes *in vivo* (45). In this model the administration of heterologous rabbit anti-PLA2R antibodies (with no data on IgG subclass) increased dose-dependently proteinuria (to the nephrotic range) with limited C3 deposition, leaving the proportional role of IgG4 predominant anti-PLA2R antibodies and membrane attack complex (C5b-9) unclear. It is conceivable that the early rise of proteinuria in experimental models of MN may be complement-independent as both anti-THSD7A and anti-PLA2R antibodies were shown to directly mediate podocyte damage in MN. However, their interaction with complement activation (if any) remains to be elucidated.

There is currently ongoing trial in anti-PLA2R antibody-positive primary membranous nephropathy with an oral inhibitor of factor B iptacopan (LNP023) directed of the alternative complement pathway (NCT04154787) and one small phase 2 safety trial with anti-MASP2 monoclonal antibody narsoplimab (NCT02682407) inhibiting complement lectin pathway.

Epitope spreading may play an important role in the severity of podocyte injury. Commonly in autoimmune diseases, primary response to immunodominant antigens may expand to another epitope on the same protein or to dominant epitopes on neighboring molecules (6, 46). It has been shown (47) that anti-PLA2R antibodies may be restricted only to the cysteine-rich (CysR) domain, or the anti-PLA2R response may also spread the C-type lectin domain 1 (CTLD1) and C-type lectin domain 7 (CTLD7) of PLA2R. Epitope spreading was shown to be associated with a more severe course of the disease.

## Genetics of MN and Anti-Podocyte Antibodies

An early GWAS in 556 European patients (48) identified two loci strongly associated with MN: the PLA2R1 gene on chromosome 2q24 and HLA-DQA1 on chromosome 6p21 with homozygosity for both alleles, increasing the risk of MN more than 78 times. A similar kind of association (allele in the HLA system likely facilitating the response against the target antigen and the risk allele of the target antigen itself) has also been described for PR3-positive ANCA-associated vasculitis (49). The strong association of three single nucleotide polymorphisms (SNPs) within PLA2R1 and one within HLA-DQA1 was confirmed in 1,112 Chinese patients with MN (50). Risk alleles were associated with the presence of anti-PLA2R antibodies and their glomerular expression. Retrospective analysis of anti-PLA2R antibodies and genotyping of DQ alleles and analysis of PLA2R1 SNPs performed in 90 prevalent patients with idiopathic MN with a 90-month follow-up demonstrated that levels of anti-PLA2R antibodies were significantly linked to DQA1^*^05:01 and DQB1^*^02:01 (51).

A recent large GWAS in 3 782 patients of European and East Asian ancestry (52) identified two other risk loci (NFKB1 and IRF4) and provided a fine mapping of PLA2R1 (rs178312151). Interestingly, this GWAS demonstrated the ancestry-specific effect of HLA alleles: DQA1^*^0501 in Europeans, DRB1^*^1501 in East Asians, and DRB1^*^0301 in both ethnicities with identified loci explaining the 32% disease risk in East Asians and 25% in Europeans. All these studies clearly showed that MN and more specifically an autoimmune response to PLA2R are strongly genetically mediated. The genetic contribution of the identified risk loci is much stronger in MN compared with IgA nephropathy, where it explains no more than 5% of the overall disease risk (53).

## Anti-Podocyte Antibodies and Non-Invasive Diagnosis of MN

As anti-podocyte antibodies are highly specific for primary MN [well-documented for anti-PLA2R antibodies, where the specificity is 98–100% (54)] it has been suggested that the diagnosis of primary MN may be assessed in patients with nephrotic syndrome, normal renal function and positive anti-PLA2R antibodies even without renal biopsy (54). However, there are several drawbacks. Anti-PLA2R antibodies should be measured with ELISA, and the reference ranges should be better established (55). Limited data are available concerning anti-PLA2R antibody specificity in non-white populations. Larger studies are required to help to define the positive predictive value of anti-PLA2R positivity as this testing is usually performed in selected patient populations with and without nephrotic syndrome and, anti-PLA2R positivity was shown to precede clinical diagnosis of MN by months to years (56). In these patients, however, anti-PLA2R positivity could predict the future development of nephrotic syndrome. As expected, increasing sensitivity can be achieved only at the expense of decreasing specificity [ <75% with a low cutoff level (57)].

The specificity of anti-PLA2R and anti-THSD7A antibodies is very high in terms of comparing patients with MN and other glomerular diseases. Both autoantibodies are also more frequent in primary MN (58); however, specificity with which anti-PLA2R positivity excludes secondary MN, especially cancer-associated MN is lower. Similarly, anti-THSD7A antibodies are highly specific for MN; however, they also do not reliably distinguish between primary and secondary MN (39).

Of note, anti-PLA2R positivity cannot be used as a non-invasive diagnostic test in patients with impaired renal function and also, in patients with suspected concomitant diabetic kidney disease or other glomerular diseases. Renal biopsies also provide an estimate of the degree of tubulointerstitial damage, strongly associated with renal outcomes and may also have a substantial impact on the response to immunosuppressive treatment. Systemic disease, although rare, namely sarcoidosis or antineutrophil cytoplasmic antibody (ANCA)-associated vasculitis, should also be considered in patients with anti-PLA2R positive antibodies. Rare necrotizing/crescentic glomerulonephritis in MN canot be also recognized without renal biopsy (32, 33).

## Anti-PLA2R and Anti-Thsd7A Antibodies and Cancer

According to a recent meta-analysis (59), the prevalence of cancer in MN is 10% (most commonly lung cancer in 26% of patients, followed by prostate cancer in 15% of patients) with a diagnosis of cancer preceding the diagnosis of MN in 20% of patients.

THSD7A is expressed by some cancers and it has been suggested that anti-THSD7A antibodies are associated with an increased risk of cancer-associated MN (60), and screening for cancer was recommended in patients with anti-THSD7A positive MN (61).

In a large Chinese study, anti-THSD7A antibodies were positive in only 2% of patients with cancer-associated MN (29). Surprisingly, 41% of patients with cancer-associated MN had anti-PLA2R1 antibodies. In another Chinese study (62), 33% of patients with cancer-associated MN were positive for both circulating anti-PLA2R antibodies and glomerular (IgG4-dominant) PLA2R staining. Although 56% of patients had THSD7A positive staining in cancer tissues, circulating antibodies were not detected. These studies suggest that anti-PLA2R antibodies may be quite commonly (and anti-THSD7A uncommonly) positive in (at least Chinese) patients with cancer-associated MN. However, it is unclear whether these findings are purely coincident, or if PLA2R antibodies participate in the pathogenesis of cancer-associated MN.

In a recent study in Caucasian patients (63), anti-PLA2R antibodies were positive in 75% of patients with idiopathic MN and in 9 out of 32 (28%, in seven patients with cancer) with secondary MN. The specificity of anti-PLA2R antibodies decreased from 100 and 94.6% compared to normal and pathological (other glomerulonephritides) controls to only 71.9% vs. secondary MN.

Antigen-specific IgG subclasses and their association with cancer were studied in patients with anti-PLA2R antibodies or anti-THSD7A antibodies (64). All patients had the most frequent IgG4 antibodies. In patients with idiopathic MN, IgG subclass distribution levels of IgG4 antibodies were similar to those of patients with malignancy-associated MN. This suggested that the pathogenesis of primary and malignancy-associated MN with either anti-PLA2R or anti-THSD7A antibodies may be similar to those in primary MN.

In the consecutive series of anti-PLA2R and anti-THSD7A negative patients 3.8% had **NELL-1** as a target antigen with segmental or incomplete IgG staining dominantly of IgG1 subclass. Out of them **33% had concurrent malignancy** which is higher proportion than in anti-PLA2R and anti-THSD7A positive patients (65).

Although cancer is more common in anti-PLA2R antibody-negative patients, diagnostic workup for cancer cannot be completely avoided, even in patients with anti-PLA2R positive antibodies (66). Elucidation of mechanisms underlying anti-podocyte autoimmunity induced by cancer warrants further research.

## Anti-Podocyte Antibodies and the Outcome of MN

Up to a third of patients with MN may develop spontaneous remission and can be spared immunosuppressive treatments, sometimes associated with serious adverse events. This is reflected by the recommendation that immunosuppressive treatment should be postponed by as many as 6 months in patients without poor prognostic signs, such as impaired glomerular filtration rate and high-grade proteinuria (9).

The chance of developing spontaneous remission was shown to be related to the titer of anti-PLA2R antibodies (67–70) and is comparatively low in patients with high titers of anti-PLA2R antibodies who could be treated with immunosuppression without unnecessary delay.

Spontaneous remission occurred significantly less often in patients with high antibody titers [38 vs. 4% in the lowest and highest tertiles (68)]. Patients with low anti-PLA2R levels had 2.72 times higher rates of spontaneous remission after a median follow-up of 2.9 years. Alternatively, high anti-PLA2R levels were associated with persistent proteinuria and a need for immunosuppressive therapy (70). Complete spontaneous remission was also more common in patients with lower anti-PLA2R levels at presentation (71).

The titer of anti-PLA2R antibodies was also closely linked to the outcome in terms of a higher risk of declining renal function during a follow-up period of 90 months (51). In another prospective study in patients with MN and positivity for anti-PLA2R antibodies with a median follow-up of 27 months, clinical endpoint (defined as an increase in serum creatinine by ≥25% and serum creatinine reaching ≥1.3 mg/dL) was reached in 69% of patients in the highest tertile of the titer of anti-PLA2R antibodies compared to only 25% of patients in the lowest tertile at inclusion into study. The study endpoints were also significantly shorter in patients with the highest compared to the lowest tertile of anti-PLA2R antibodies [17.7 vs. 30.9 months (72)]. In another study, however, long-term renal survival (at 5, 10, and 15 years) was high in all tertiles of anti-PLA2R antibodies (97, 93, and 89%, respectively), and was only related to the severity of proteinuria (70).

Poor renal outcomes in patients with anti-PLA2R antibodies may also be associated with epitope spreading of anti-PLA2R autoimmune response with less frequent nephrotic syndrome at presentation, a higher rate of spontaneous remission, and lower risk of progression to renal failure in patients with anti-PLA2R antibodies restricted to cysteine-rich domain of the PLA2R, compared with patients with concomitant positivity of antibodies to C-type lectin domain 1 and C-type lectin domain 7 (47). Epitope spreading is strongly correlated with titers of anti-PLA2R antibodies and a lower rate of remission at 6 months (73).

Epitope spreading is thus an important independent predictor of poor outcomes, and patients with epitope spreading should be treated without delay with higher doses of rituximab. As epitope-specific assays for anti-PLA2R are not available, epitope spreading cannot be evaluated in clinical practice and we must rely on the correlation of epitope spreading with anti-PLA2R titers (73). Similar to patients with high titers of anti-PLA2R antibodies, patients with high titer of anti-THSD7A antibodies also have poor clinical outcomes (30).

In summary, low baseline and decreasing anti-PLA2R antibody levels strongly predict spontaneous remission, thus favoring conservative therapy. Conversely, high baseline or increasing anti-PLA2R antibody levels are associated with nephrotic syndrome and progressive loss of kidney function, thereby encouraging prompt initiation of immunosuppressive therapy (58).

## Antibodies and Transplantation

Up to 40–50% of patients with primary MN (if untreated) may finally develop end-stage renal disease. MN may recur in transplant in 30–45% of patients with recurrence being more frequently reported by centers performing regular protocol biopsies (74). Anti-PLA2R antibodies are detected in 70–80% of patients with recurrence of MN in the renal allograft (75). The risk of recurrence is higher in patients with positive anti-PLA2R antibodies before transplantation (76, 77) and with anti-PLA2R antibody staining in renal allografts (77).

Using cutoff levels of circulating anti-PLA2R antibodies of 45 U/mL, the recurrence of MN was predicted with a sensitivity and specificity of 85% and a negative predictive value of 92%. Anti-PLA2R levels and recurrence of MN in the allograft were shown to be genetically mediated [associated with HLA DQA1^*^ 05:01/05 and DQB1^*^ 02:01 (77)]. In another study (78), anti-PLA2R antibodies were positive prior to transplantation in 83% of patients who experienced recurrence.

In the patient with anti-THSD7A-associated MN, who reached end-stage renal failure and was subsequently transplanted with early recurrence of MN in the graft, the anti-THSD7A antibodies were detectable both before and after transplantation. The staining for THSD7A was also documented in the allograft, suggesting the pathogenic role of anti-THSD7A antibodies (43).

## Anti-Podocyte Antibodies and Activity of MN

Titers of anti-PLA2R antibodies are strongly correlated with the clinical activity of the disease and proteinuria (67, 68, 79). This is demonstrated by the antibody titers in patients with active disease compared with patients with partial or complete remission (51). The reemergence of circulating antibodies predicts disease relapse (58, 80).

Titers of anti-THSD7A also decrease (or the antibody completely disappears) with the development of remission and once again reappear (increase) with the relapse of the disease (29). High titers of anti-THSD7A are also correlated with the activity of the disease. However, due to the disease sparsity, the evidence is less convincing than that of the PLA2R antibodies (30). Regular measurement of the levels of anti-PLA2R and anti-THSD7A antibodies may thus be used for monitoring the activity of the disease.

## Anti-Podocyte Antibodies and Response to Treatment

Patients with primary MN are currently treated with either alkylating cytotoxic drugs, calcineurin inhibitors, B-cell depleting monoclonal antibody, or rituximab. Alkylating cytotoxic drugs are effective, but their use is associated with significant adverse events. The use of calcineurin inhibitors is associated with a high relapse rate on their tapering and withdrawal.

Reduction in the levels of circulating anti-PLA2R antibodies preceded by several months and predicted a decrease in the proteinuria in rituximab-treated patients with MN (68, 81–83).

In 133 adult patients with anti-PLA2R positive MN, the antibody levels decreased after 3 months of immunosuppressive treatment by 81% and proteinuria by 39% (72). Patients who developed remission 12 months later had significantly lower levels of anti-PLA2R at baseline compared to patients with no remission. Moreover, patients with high levels of anti-PLA2R antibodies achieved remission later than patients with low levels. The antibody levels remained elevated in patients who did not achieve remission of proteinuria.

In another study, 84 out of 132 rituximab-treated patients with idiopathic MN achieved complete or partial remission during a median follow-up of 30.8 months and 25 relapsed after remission (80). In 81 patients with positive anti-PLA2R antibodies, remission was strongly predicted by a lower antibody titer prior to treatment. These patients had complete antibody depletion 6 months after the initiation of rituximab treatment. In all patients, depletion of anti-PLA2R antibodies preceded complete remission. Early reduction of the titer of anti-PLA2R antibody by 50% was associated with a decrease in proteinuria by 50% by 10 months. In this study, response to rituximab treatment was not related to the polymorphisms of both PLA2R1 and HLA-DQA1.

The role of immunologic remission (depletion of anti-PLA2R antibodies) in MN was recently demonstrated in three randomized controlled trials, GEMRITUX (84), MENTOR (85) and STARMEN (86).

In the GEMRITUX trial (84), 75 patients with biopsy-proven MN and nephrotic syndrome were randomized either to rituximab or non-immunosuppressive antiproteinuric treatment. Anti-PLA2R depletion was achieved at 6 months in 50% of patients treated with rituximab treated and only in 12% of patients treated with non-immunosuppressive antiproteinuric treatment. Although at 6 months there was no significant difference between both limbs in combined complete and partial remission (35.1 vs. 21.1%), at the end of observational phase (after median follow-up of 17 months) remission rates were significantly higher in patients treated with rituximab compared with patients treated with non-immunosuppressive antiproteinuric treatment (64.9 vs. 34.2%). Early responses in terms of anti-PLA2R levels predicted the later development of clinical remission.

In the MENTOR trial (85), 130 patients were randomized to rituximab or cyclosporine. Early response (at 12 months) to either rituximab of cyclosporine was similar (complete and partial remission induced in 60% vs. 52% of patients, respectively). However, at 24 months, complete or partial remission was much more frequent in rituximab- than in cyclosporine-treated patients (60 vs. 20% of patients, respectively). It should be, however, stressed, that cyclosporin was withdrawn already at 12 months, so these data reflect namely the risk of early relapse after cyclosporine withdrawal. Importantly, the decline of anti-PLA2R antibodies was faster and of greater magnitude and duration in rituximab vs. cyclosporine. This again demonstrated that immunological remission (depletion of anti-PLA2R antibodies) was indispensable for clinical remission.

In a recently published STARMEN trial (86) which randomized 86 pts with membranous nephropathy at high risk or progression to either cyclical alternating treatment with corticosteroids and cyclophosphamide or tacrolimus followed by single dose of rituximab complete or partial remission of nephrotic syndrome at 24 months was reached in 83.7% of patients treated with corticosteroid-cyclophosphamide and in only 58.1% of patients treated with tacrolimus and rituximab. Immunological remission (depletion of anti-PLA2R antibodies) was achieved at 3 and 6 months in 77 and 92%, respectively, of pts in corticosteroid-cyclophosphamide limb compared to 45 and 70% of patients in the tacrolimus-rituximab limb–in fact, it reflects the efficacy of tacrolimus only as rituximab was given only at 6 months). Corticosteroid-cyclophophamide treatment thus induced compared to tacrolimus-rituximab more often both complete and partial remission and induced imunological remission more quickly.

In a small open-label study belimumab (monoclonal antibody against B lymphocyte stimulating factor BAFF-BLyS) decrease of proteinuria was preceded by the decrease of the titer of anti-PLA2R antibodies (87). There is currently ongoing clinical trial (REBOOT, NCT03949855) in anti-PLA2R positive membranous nephropathy comparing the combination of belimumab with rituximab with rituximab only.

Rituximab was also shown to reverse epitope spreading in almost 60% of treated patients (88). In the GEMRITUX trial (84), epitope spreading was strongly correlated with the titer of circulating anti-PLA2R antibodies, and reversal of epitope spreading was observed in 10 out of 17 patients (58%) with epitope spreading at baseline treated with rituximab.

Available data suggest that the early decrease in levels of anti-PLA2R antibodies is a predictor of later clinical remission, which may develop slowly within more than 1 year. As the chance to develop clinical remission is very high in patients with treatment-induced depletion of anti-PLA2R antibodies, no further immunosuppression (e.g., maintenance treatment with rituximab) may be indicated. Instead, monitoring and waiting may be a safer and more reasonable approach (2, 58, 80).

Alternatively, a decrease in proteinuria without an immunological response (as in some patients treated with cyclosporine) should not be interpreted as real remission. There is a high risk of relapse when treatment (most frequently cyclosporine) is withdrawn. Persistence of high titer of anti-PLA2R antibody is a sign of ongoing immunologic activity (and possibly ongoing podocyte damage) despite lower proteinuria. Thus, the efficacy of any immunosuppressive treatment should be assessed based on the induction of immunologic remission. The reappearance of anti-PLA2R positivity and/or an increase in the previously decreased titer of anti-PLA2R antibody is a clear sign of the impending relapse of MN, sometimes preceding the rise of proteinuria. Serial measurements of anti-PLA2R antibodies (monthly, or at least bi-monthly) during and at the end of the cycle of immunosuppressive treatment may help personalize the treatment of MN (2).

Response to treatment in anti-PLA2R positive patients is characterized by a rapid decrease in the titer of anti-PLA2R antibodies. The response to treatment is worse in patients with high levels of anti-PLA2R antibodies before the start of treatment. Levels of anti-PLA2R antibodies are independent predictors of response to treatment (in terms of achieving remission). Levels of anti-THSD7A antibodies are also related to the response to immunosuppressive treatment. In a subgroup of patients with serial titers, persistently elevated anti-THSD7A autoantibodies were observed in patients who wither did not respond to treatment or achieve remission (30). Hopefully, our experience will be soon extended to patients with anti-NELL-1 antibodies (18).

## Conclusions

Identification of anti-podocy antibodies elucidated the pathogenesis of primary MN and enabled its non-invasive diagnosis. Titers of anti-podocyte antibodies predict the renal outcome and response to treatment and help personalize the immunosuppressive therapy. Persistent remission and prevention of progression to end-stage renal disease cannot be achieved without suppression of the podocyte-directed autoimmune response. With the identification of several further minor podocyte antigens classification of primary membranous nephropathy will be based on the type of autoantibody, its IgG subclass and potential to activate complement resulting possibly in the future preference of either B cell- or complement-targeted therapeutic approaches.

## Author Contributions

Both authors contributed equally to the review of the available literature and preparing the manuscript.

## Conflict of Interest

The authors declare that the research was conducted in the absence of any commercial or financial relationships that could be construed as a potential conflict of interest.
